# Prediction of Spontaneous Regression of Cervical Intraepithelial Neoplasia Lesions Grades 2 and 3 by Proteomic Analysis

**DOI:** 10.1155/2014/129064

**Published:** 2014-06-15

**Authors:** Kai-Erik Uleberg, Irene Tveiterås Øvestad, Ane Cecilie Munk, Cato Brede, Bianca van Diermen, Einar Gudlaugsson, Emiel A. M. Janssen, Anne Hjelle, Jan P. A. Baak

**Affiliations:** ^1^Norconsult AS, Section 355 QA Service, P.O. Box 216, NO-4503 Mandal, Norway; ^2^Former International Research Institute of Stavanger (IRIS), P.O. Box 8046, 4068 Stavanger, Norway; ^3^Pathology Department, Stavanger University Hospital, P.O. Box 8100, 4068 Stavanger, Norway; ^4^Department of Gynecology and Obstetrics, Stavanger University Hospital, P.O. Box 8100, 4068 Stavanger, Norway; ^5^Department of Medical Biochemistry, Stavanger University Hospital, P.O. Box 8100, 4068 Stavanger, Norway; ^6^Mediteam AS, Sjøveien 34, 4315 Sandnes, Norway; ^7^The Gade Institute, University of Bergen, P.O. Box 1400, 5021 Bergen, Norway

## Abstract

Regression of cervical intraepithelial neoplasia (CIN) 2-3 to CIN 1 or less is associated with immune response as demonstrated by immunohistochemistry in formaldehyde-fixed paraffin-embedded (FFPE) biopsies. Proteomic analysis of water-soluble proteins in supernatants of biopsy samples with LC-MS (LTQ-Orbitrap) was used to identify proteins predictive of CIN2-3 lesions regression. CIN2-3 in the biopsies and persistence (CIN2-3) or regression (≤CIN1) in follow-up cone biopsies was validated histologically by two experienced pathologists. In a learning set of 20 CIN2-3 (10 regressions and 10 persistence cases), supernatants were depleted of seven high abundance proteins prior to unidimensional LC-MS/MS protein analysis. Mean protein concentration was 0.81 mg/mL (range: 0.55–1.14). Multivariate statistical methods were used to identify proteins that were able to discriminate between regressive and persistent CIN2-3. The findings were validated in an independent test set of 20 CIN2-3 (10 regressions and 10 persistence cases). Multistep identification criteria identified 165 proteins. In the learning set, zinc finger protein 441 and phospholipase D6 independently discriminated between regressive and persistent CIN2-3 lesions and correctly classified all 20 patients. Nine regression and all persistence cases were correctly classified in the validation set. Zinc finger protein 441 and phospholipase D6 in supernatant samples detected by LTQ-Orbitrap can predict regression of CIN2-3.

## 1. Introduction

Among cancers affecting women, cervical cancer has the second highest occurrence worldwide, with an incidence in 2008 of 529,800 cases (14.5% in developed countries and 85.5% in developing countries) and 275,000 estimated deaths [1]. Infection of cervical epithelial cells with high risk human papillomavirus (HPV) is the most important risk factor for development of cervical cancer, as first highlighted by zur Hausen [2]. Noninvasive cervical intraepithelial neoplasia (CIN) precedes the development of invasive cancer and may progress from CIN2-3 to (micro)invasive cancer in 10–25 years on average [3].

Three CIN grades (CIN1, CIN2, and CIN3) are recognized by the World Health Organization to distinguish the degrees of epithelial abnormality and are associated with increasing risks for invasive cancer development. A CIN lesion is, however, not a static event but a dynamic process that can persist and progress but also spontaneously regress [4, 5]. If left untreated, 5–30% of all CIN2-3 lesions (confirmed by a histological punch biopsy) will develop invasive cancer [6]. On the other hand, without cone excision, as many as 32–43% of CIN2-3 lesions will regress spontaneously [7]. Nevertheless, in many countries including Norway, all punch biopsy-confirmed CIN2-3 lesions are usually treated with diathermic cone excision, a fairly aggressive therapy which can have serious adverse side effects [8]. The most serious late-complication is cervical insufficiency which can lead to late abortion and preterm delivery during the second and early third trimester of a future pregnancy [9, 10].

Until recently, regression of CIN2-3 lesions could not be effectively predicted. However, research on functional biomarkers like pRb, p53, and cytokeratin 13/14 has proven to be helpful in predicting regression, especially when combined with local immune response and HPV genotype [11–14]. Furthermore, combined Ki67 and pRb expression can predict which CIN1 lesions will progress to CIN3 [15]. Aggregated information provided by such epithelial biomarkers and local cellular immune response in the microenvironment of CIN2-3 lesions supports prediction of regression/persistence/progression and may result in even more accurate CIN treatment, [16] as well as reducing overtreatment of patients with CIN2-3 lesions.

Unfortunately, the procedures used to obtain formalin-fixed, paraffin-embedded (FFPE) tissue from biopsies irreversibly degrade water-soluble proteins. A protein collection method for small punch biopsy samples that could represent not only the cellular response but also proteins from the cervical neoplasia microenvironment and intracellular compartments may further help define the biology of CIN lesions' dynamic behaviour. We have recently described a method that can preserve and extract water-soluble proteins from punch biopsies [17], how a panel of 3 peaks from SELDI-TOF protein profiles can be used to differentiate normal tissue from CIN tissue samples, and that a discrimination between CIN2 and CIN3 lesions could be obtained using cytokeratin 2 [18].

In the present study we analysed protein samples from CIN2-3 lesions with known regression/persistence status. We have used both SELDI-TOF MS and bottom-up shotgun proteomics [19] approach utilizing nanoflow liquid chromatography coupled to a LTQ-Orbitrap mass spectrometer. The goal was to identify proteins that could be used in prediction of regression or persistence in CIN2-3.

## 2. Materials and Methods

### 2.1. Study Population

This study is a subproject from a larger prospective study, approved by the Regional Medical Ethics Committee of Helse Vest, Norway, the Norwegian Data Inspection, and the Health Directorate of Norway, numbers 33.06, 17185, and 07/330. Healthy women aged 25-40 years, with cytological abnormal smears were followed by cervical biopsy and later cone excision. In total, 170 patients with first time onset of CIN2-3 were included from January 2007 to December 2008. The interval between punch biopsy and cone excision was standardized at median 113 days (range: 100–126). This interval was chosen in view of a previous study, which showed that CIN2-3 patients with more than 9-week punch-cone interval have a much higher chance on regression than those with <9-week interval [5]. Regression was defined as CIN1 or less in cone histology and regression rate was 22% (38/170). All patients included in this study were treated according to the national Norwegian population screening quality guidelines [20].

In our cohort of patients we first analysed whether proteins and peptides detected by proteomic LC-MS (LTQ-Orbitrap) could distinguish between CIN2-3 lesions, with and without later regression. Of the 170 patients with cervical punch biopsy samples, a random subset of 20 patients with cervical intraepithelial neoplasia lesions (see below for reviewing details), 10 CIN2 (5 with regression and 5 with persistence) and 10 CIN3 (5 with regression and 5 with persistence), were selected and defined as the learning set. The histological diagnosis was set by two experienced pathologists. The samples were selected so that the whole sampling period was covered and the protein concentration was as close as possible to the average for the whole data set. In a second validation study, another 20 CIN2-3 patients (10 CIN2 cases, 5 with regression and 5 with persistence, and 10 CIN3, 5 with regression and 5 with persistence, defined as the validation set) were selected to test the prognostic value of the proteins found in the learning set.

For the SELDI-TOF study, the sample set from a former investigation was used [21]. These samples were statistically reanalysed with regard to the regression/persistence status. Thus 2 replicates of each of the 5 regression and 40 persistent CIN2-3 samples were included for this part of the study.

### 2.2. Sample Collection

After colposcopy, punch biopsies and endocervical curettage were taken from the transformation zone and eventually premalignant mucosa. One or two biopsies were immediately placed in polystyrene tubes (Sarsted, Numbrecht, Germany) containing 5 mL RPMI-1640 (Gibco, Carlsbad, USA) tissue culture medium. The biopsies were kept in the tissue culture medium for 24 hours at 4°C before the supernatants were collected, split into aliquots of 500 *μ*L, and stored at −80°C until analysis. Immediately after sample collection, an additional set of biopsies were stored in 4% buffered formaldehyde according to standard procedure.

### 2.3. Pathology

As described before [17] after 24 hours of incubation in RPMI-1640 medium at 4°C, the biopsies were routinely fixed in buffered 4% formaldehyde, embedded in paraffin, cut at 4 *μ*m, and stained with hematoxylin, eosin, and safran (HES) for routine histological examination. P16 and Ki67 (MIB-1) immunohistochemical (IHC) staining were used to confirm the diagnosis. All HES and IHC sections of the 170 biopsies were reviewed by two independent pathologists, who also used the p16 and Ki67 immunohistochemical information. The participating pathologists were blinded to the original routine clinical findings, histopathological diagnosis, and follow-up. In case of discrepancies the cases were reviewed and diagnosed on a double-head microscope by the two pathologists (Einar Gudlaugsson and Jan Baak) and a consensus diagnosis was obtained.

### 2.4. ProteinChip SELDI-TOF MS Analysis

Protein concentrations were assessed using the Bradford methodology. Samples were subjected to SELDI-TOF MS profiling according to the manufacturer's instructions (Ciphergen Biosystems, Fremont, CA, USA). The biopsy supernatants were diluted 1 : 5 with 50 mM sodium acetate (pH 4.3) and then bound to a CM10 ProteinChip array. They were incubated for two hours at room temperature on a platform shaker and then washed twice with 50 mM sodium acetate buffer, followed by two washes of 1 *μ*L energy absorbing molecule (=EAM) solution (consisting of 50% saturated synaptic acid dissolved in 50% acetonitrile and 0.5% trifluoroacetic acid). Two replicates were prepared on different CM10 ProteinChips by two different technicians on two different days. The time-of-flight spectra were generated on the Protein Biological System II mass spectrometer reader (Ciphergen Biosystems, Fremont, CA, USA), using a laser intensity of 170 and a detector sensitivity of seven. Readings were optimized for low molecular weight (2–20 kDa). External mass calibration was performed daily.

### 2.5. SELDI-TOF MS Data Analysis

The SELDI-TOF MS data analyses were performed in three steps: (1) peak detection, (2) selection of peaks with the highest discriminatory power, and (3) building a multivariate model based on the selection in step (2). The peak detection was done using the Ciphergen Seldi software version 3.2 after internal and external mass calibration followed by normalization (total ion current, TIC, intensity) of all spectra as one group. The mass range from 2000 to 20000 Da contained the majority of the peptides/proteins in the samples and was selected. Masses less than 2000 Da were excluded as these are known to contain adducts and artifacts from the EAM solution and other chemical contaminants. The peak detection includes baseline subtraction, calibration of mass accuracy, and automatic peak detection. Each spectrum was then assigned to one of three groups, normal, regression, or persistence. To select peaks with the highest discriminatory power, the Biomarker Wizard (Ciphergen) was used for peak detection and clustering of all the spectra. This was done using a signal-to-noise (s/n) ratio of 5 and 15% of all spectra for the first pass detection and clustering and an s/n ratio of 2 for the second pass. The cluster results were then imported into SPSS (v17, SPSS Norway AS, Oslo, Norway), CART (Salford, San Diego, CA, USA), and MedCalc (MedCalc Software, Mariakerke, Belgium) for binary logistic regression analysis.

### 2.6. Immunoaffinity Depletion

The preparation and use of the immunoaffinity column is described in [17]. To deplete samples of the 7 high abundance proteins, 100 *μ*L of RPMI supernatant was diluted with 100 *μ*L Tris-buffered saline (TBS, 0.1 M TRIS-base containing 0.1 M NaCl, pH 8.0), and the solution was injected into a TBS solution with a flow of 0.2 mL/min. The nonretained proteins were trapped on a 4 mm × 2.0 mm (inner diameter, i.d.), C_18_ security guard cartridge with 300 Å pore size (Phenomenex, Teknolab, Norway) and were eluted by backflushing the security guard cartridge with ethanol at a flow of 0.3 mL/min. The affinity column was washed using 0.1 M glycine at pH 2.5 with a flow of 1.2 mL/min. Both columns were reequilibrated with TBS at a flow of 0.2 mL/min for 5 minutes. The pH adjustments were done using 6 M HCl.

### 2.7. Protein Digestion and Sample Cleanup

After evaporating the ethanol phase containing the nonretained protein fraction using vacuum centrifugation (Eppendorf Concentrator 5301, VWR, Norway), 100 *μ*L 50 mM ammonium bicarbonate pH 8 was added to the samples. 1 *μ*L of  1 M dithiothreitol (DTT) was added to reduce the proteins. 5 *μ*L of 1 M iodoacetamide (IAA) was then added to alkylate the proteins followed by 5 *μ*L of DTT to stop the alkylation process. For each of these steps, 45-minute incubation time was used. One *μ*g trypsin (Promega) was added, and the samples were kept at 37°C for 18 hours. After digestion with trypsin the samples were purified and concentrated using a C_18_ ZipTip (Millipore, Norway) procedure. The ZipTips were conditioned by aspirating 30 *μ*L acetonitrile five times and equilibrated with pulling 30 *μ*L 0.1% formic acid (FA) in MilliQ water five times through the stationary phase. Approximately 10 *μ*L of the 0.1% FA solution was left above the stationary phase to avoid drying it. Each sample solution was applied on top of the stationary phase using a pipette and then pushed through the tip using air pressure from the pipette plunger. More sample solution was added when approximately 20 *μ*L of the liquid remained so that the whole volumes of the samples were pushed slowly through the ZipTip. Washing was done by aspiring 30 *μ*L of 0.1% FA five times. Elution of the peptides was done in a total volume of 30 *μ*L of 80 : 20 (v/v) acetonitrile : MilliQ water by aspiring 10 *μ*L of this solution 10 times through the stationary phase. The organic phase was then evaporated using vacuum centrifugation and, to the residual solution, 20 *μ*L 0.1% FA was added prior to the LC-MS/MS analysis.

### 2.8. LC-MS/MS Analysis

A Dionex Ultimate 3000 nanoflow HPLC equipped with a 300 *μ*m (i.d.) × 0.5 cm length Acclaim PepMap 100 C_18_ trap column and a 75 *μ*m (i.d.) × 15 cm Acclaim PepMap 100 C_18_ analytical column (Dionex) was used with a LTQ-Orbitrap hybrid mass spectrometer (Thermo Scientific). 5 *μ*L of the tryptic digests was injected onto the trap column using 0.1% formic acid (VWR) in MilliQ-water at a flow of 2 *μ*L/min. The separation was done using a gradient from 2.5% to 64% acetonitrile in 0.1% FA over 180 minutes at a flow of 300 nL/min. A 10-minute postinjection delay and a 20-minute column reequilibration time were used. The electrospray interface was a PicoTip emitter (SilicaTip, New Objective) with a 10 *μ*m tip without coating. The electrospray voltage was set to 1 kV. No sheath gas was used. The mass spectrometer was used in positive mode. Full scans were performed in the Orbitrap using the *m*/*z* range from 200 to 2000. Data dependent MS/MS scans were performed in the LTQ for the five most abundant masses with *z*≥ 2 and intensity higher than 10,000 counts. Dynamic exclusion for 3 minutes after fragmentation of a given *m*/*z* value four times was used. Collision induced dissociation (CID) was used with a collision energy of 35%, activation Q setting of 0.400, and 30 ms activation time for MS. Calibration of the mass spectrometer was done weekly using the calibration solution recommended by Thermo Scientific.

### 2.9. Bioinformatic Data Analysis

The raw data files were analysed using the Proteome Discoverer 1.0 (Thermo Scientific) with the Sequest algorithm to search against the* Homo sapiens* (Tax.id: 9606) database at NCBI (531420 sequences) with trypsin as digestion enzyme allowing for 2 missed cleavages. All files were also searched against the human papillomavirus database (Tax.id: 10566) at NCBI (1615 sequences). Precursor ion tolerance was set to 10 ppm, and fragment ion mass tolerance was set to 0.8 Da. Oxidation (M) was set as a dynamic modification and carbamidomethyl (C) was set as a static modification due to the use of DTT and IAA. Phosphorylation (STY) was set as a dynamic modification. A high significance peptide confidence filter was set in Proteome Discoverer (PD) from Thermo, which means that peptide identifications are filtered based on the following combination of charge and Xcorr factor: 1.9 (*z* = 2), 2.3 (*z* = 3), and 2.6 (*z*≥ 4). Additional information for proteins was obtained from the UniProt database entry.

Protein identifications were accepted using one peptide when certain requirements were fulfilled: the Sequest Xcorr factor with regard to charge had to be fulfilled according to the high significance criteria in PD. The peptide had to contain at least 7 amino acids and have at least three consecutive b- and y-ions in the MS2 spectra [22], and it should occur minimum three times in the same sample. In addition, for proteins with only one identified peptide sequence, the peptide sequences were submitted for a BLAST search against the Uniprot* Homo sapiens* database (htttp://www.uniprot.org/) to confirm that the identification matched the NCBI identification. For proteins listed as unnamed in the NCBI database, the ID mapping tool at UniProt was used to see if the protein was listed with a more descriptive annotation in this database. Only proteins identified in at least 30% of the samples in one of the groups (regression/persistence) were included in the remaining work. Spectral count (SPC) results for the identified peptides were obtained and used for normalization (see (1)) after increasing all numbers with 1 to avoid zeros. Consider

(1) SPC  NormProtein  x, Sample  x,  Group  y =SPCProtein  x,Sample  x×Sum  SPCSample  xAvg.  SPCGroup  y.

### 2.10. Multivariate Analysis of the LC-MS/MS Data

The normalized SPC results were imported into Sirius (version 8.1, Pattern Recognition Systems, Bergen, Norway) to perform a partial least-squares discriminant analysis (PLS-DA) using a target projection component, calculations of selectivity ratios (SR), plotting of SR-values, and a discriminating variable (DIVA) test. A binary response variable was added to the SPC dataset to assign all samples to one of the two groups (i.e., regression = 0 and persistence = 1), making it a supervised method. Partial least-squares discriminant analysis (PLS-DA) is a suitable method when the within group variance is comparable or dominant compared to the between group variance [23]. The maximum group discrimination from a PLS-DA model can be represented by a target projection (TP) component that is obtained by combining all PLS components into this single TP component using a latent variable projected onto the response variable [24]. A score value from the target projection model is calculated for each object (sample) with regard to the group variable. A selectivity ratio (SR) plot resembles a spectrum and is a plot of the ratio of explained variance to unexplained variance for each variable [25], where one variable in this case is an identified protein. Variables with high selectivity ratios have high discriminating ability between the two groups. The discriminating variable (DIVA) test is a nonparametric test suitable for small sample sets with group heterogeneity [24]. A correct classification rate (CCR) value is calculated for each variable and will vary between 50% for a variable that provides random classification of the samples and 100% for a variable that gives a complete separation of the two groups. The SR and CCR are closely related in that higher SR should give higher CCR. The DIVA test provides a means of setting boundaries for the selectivity ratio to identify the important discriminating variables (proteins in this case) for a given CCR. More in depth theoretical explanations of all these methods can be found in [23–25]. The model was cross-validated by leaving out a large percentage of the individual samples from both sets in two cross-validation steps. In an outer loop, 20% of the samples were kept out at a time for an external validation. This was repeated 5 times so that all samples were kept out once. In the inner loop, 25% of the samples were kept out at a time, and this was repeated four times to keep out all samples once.

The normalized spectral count data were also imported into SPSS (version 18, SPSS, Oslo, Norway) for a binary logistic regression analysis and CART (Salford, San Diego, CA, USA) for a classification and regression tree analysis, both used as supervised in the sense that a group variable (regression or persistence) was added. The continuous variables were divided into two different subgroups, using a threshold value assessed by receiver-operating curve (ROC) analysis (MedCalc Software, Mariakerke, Belgium).

## 3. Results

The median age of the patients at inclusion was 29.7 years (range: 25–40), the interval between punch biopsy and cone excision was median, 113 days, and the mean protein concentration of the selected RPMI samples, measured by Bradford, was 0.81 mg/mL (range: 0.55–1.14). The age, punch-cone excision interval, and protein concentration of the RPMI samples of each of the three groups of patients studied (i.e., LC-MC/MS learning set, validation set, and the SELDI-TOF set) were consistent with the overall cohort from which our samples were selected and therefore can be regarded as representative.

### 3.1. SELDI-TOF MS Results

**Figure 1:**
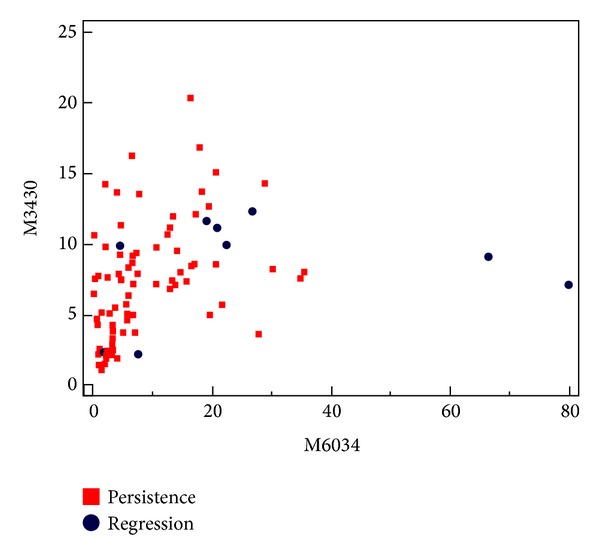
Scatter plot of the SELDI-TOF MS results for the peaks with *m*/*z* 6034 and *m*/*z* 3430 (samples: *n* = 5 regression and *n* = 40 persistence, 2 replicates of each). The figure illustrates that no discrimination is obtained between the regression and persistence groups using these results.

A total of 40 peaks were detected in the SELDI-TOF spectra using the criteria described in Section 2.5. The development of a binary logistic regression model resulted in one protein peak (*m*/*z* 6034) having the best discriminatory power between the regression and persistence samples of the 40 peaks in this dataset.  Figure 1 is a scatter plot showing this peak plotted against one of the peaks found as discriminatory between normal and CIN2-3 tissue in the previous study [21]. The figure shows that this SELDI-TOF peak in fact could not discriminate between CIN2-3 lesions with regression and persistence.

### 3.2. LC-MS/MS Results

The samples were subjected to depletion of 7 high abundance proteins followed by tryptic digestion and unidimensional LC-MS/MS analysis. Using the high significance peptide confidence filter in Proteome Discoverer and the identification criteria for proteins with only one peptide, a total of 165 protein identifications were included (all listed in Table 1 and more detailed in Supplementary Tables 1 and 2, see Supplementary Material available online at http://dx.doi.org/10.1155/2014/129064): 57 of these were identified with two or more unique peptides and the others with only one unique peptide. Although peptides from human papillomavirus proteins were detected in all samples, none of them gave acceptable protein identification.

**Figure 2:**
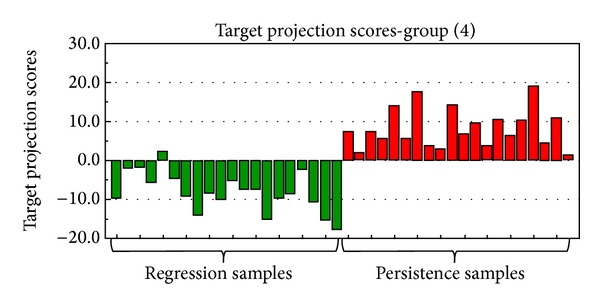
Score plot of discrimination between the regression group and persistence group after the target projection analysis.

Figure 2 shows a plot of the target projection score results from the complete dataset. The discrimination between the regression and persistence group is 95% since all persistence samples have score values with a positive sign, and 19 out of the 20 regression samples have a negative score value.

**Figure 3:**
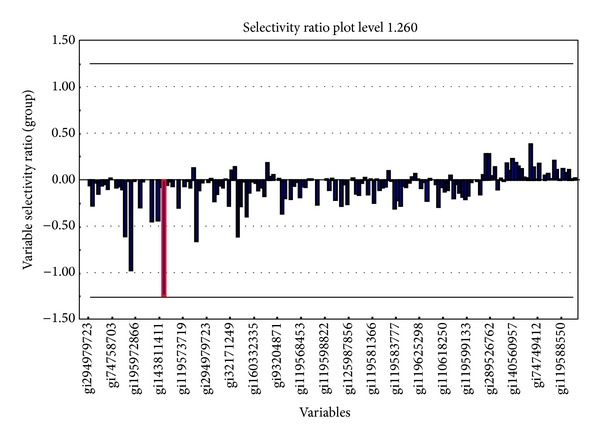
Selectivity ratio plot for all identified proteins. The selectivity ratio limit for a 90% mean corrected classification rate is shown as horizontal lines. The zinc finger protein 441 is marked with red square box.

Figure 3 shows a selectivity ratio plot for all the identified proteins resulting from doing a DIVA test with 90% correct classification rate set as an objective goal. This resulted in a selectivity ratio of ±1.26 as the limit for a variable to be significant in discriminating the groups. These limits are shown as solid horizontal lines in the figure.

Only one protein, the zinc finger protein 441 (ZNF441) (gi308153532) (red box), had a selectivity ratio of 1.26.

The CART analysis of the learning set resulted in a two-node model in which the ZNF441 was used as the primary group discriminator and, “similar to CG12314 gene product”, as the second most contributing discriminator. This protein was identified with one peptide (RVLITGSLNWTTQAIQNNR, precursor *m*/*z*: 2265.1714 Da, charge: +2). A Blast search against the UniProt human database with this sequence gave only one hit, phospholipase D6 (PLD6) (UniProt identifier: Q8N2A8). A search using the ID-mapping tool at the UniProt website gave no results. However, a UniProt Blast search for the complete sequence from the NCBI entry resulted in a unique hit with 100% identity score, PLD6.

**Figure 4:**
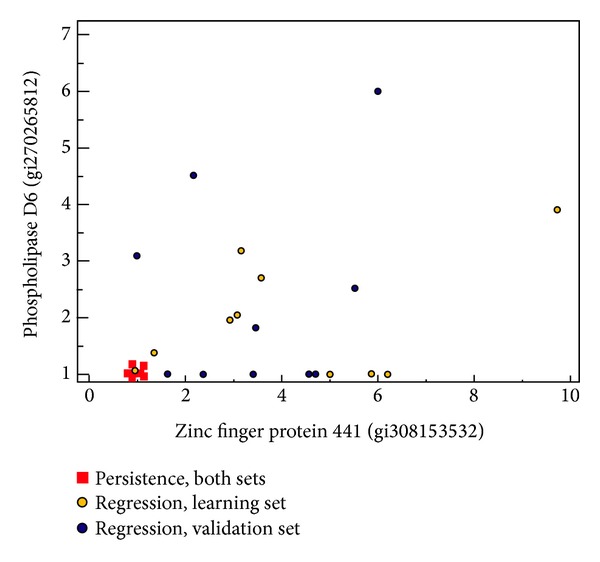
Scatter plot of the spectral count data for the zinc finger protein 441 and phospholipase D6 protein showing the discrimination between regression and persistence samples. The regression samples are shown further subdivided in learning (yellow circles) and validation (blue circles) sets.

Figure 4 shows a scatter plot using the spectral count results for these two proteins of the two sets and illustrates the discrimination obtained.

The binary logistic regression model also resulted in ZNF441 having highest discriminatory power (results not shown).

ZNF441, identified using one highly significant peptide (QCGKALSHLKSFQR), was found in 10 and 9 of the 10 regression samples in the learning and validation set, respectively, and in none of the persistence samples. The PLD6 protein was also identified using only one high significance peptide and occurred in 7 and 5 of the 10 regression samples in the two sets.

**Figure 5:**
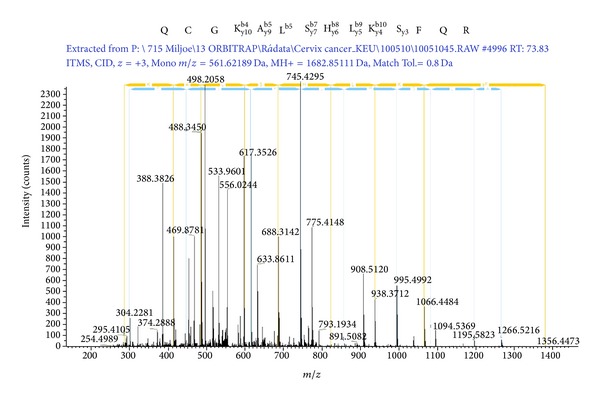
MS2 spectrum and amino acid sequence for the identified peptide from zinc finger protein 441. The y- and b-series are also shown.

Figure 5 shows the peptide sequence, the MS2 spectrum, and the y- and b-series for the ZNF441 peptide.

A Blast search using the peptide sequence against the human UniProt database gave the two ZNF441 isomers as the only proteins with a 100% sequence similarity and the best scores from the search. ZNF441 (gi308153532, UniProt: Q8N8Z8) is a nuclear protein with 693 amino acids which belongs to the Krueppel C_2_H_2_-type zinc finger protein family, and it contains 19 C_2_H_2_-type zinc fingers and 1 KRAB-domain (UniProt entry).

ROC curve analysis showed that the optimal threshold for both ZNF441 and PLD6 was ≤1 versus >1. Using these thresholds, all regression and persistence cases of the learning set were correctly classified. In the validation set, 9 of the 10 regression and all 10 persistence cases were correctly classified. Figure 4 illustrates the power of the two proteins to distinguish between regressive and persistent CIN2-3 lesions for all cases in both the learning and the test set.

## 4. Discussion

This study describes the results from analysis of three different datasets regarding regression or persistence of CIN2-3 lesions: one dataset from SELDI-TOF MS and two datasets from LC-MS/MS analysis.

For the SELDI-TOF MS study, supernatants from a total of 45 patient samples (5 CIN2-3 with regression and 40 CIN2-3 with persistence) were analysed. One discriminatory peak was found by developing a binary logistic regression model using the SELDI-TOF MS dataset, but no discrimination between CIN2-3 lesions with regression or persistence could be obtained. Other binding conditions for the CM10 chip could have been used, as well as other chip types, but this was not pursued further as obtaining protein identification from a SELDI-TOF MS peak proved challenging.

LC-MS/MS analysis was much more promising than SELDI-TOF. All three multivariate statistical methods applied on the normalized spectral count results gave the same result, indicating that ZNF441 can discriminate between regressive and persistent CIN2-3 lesions. To our knowledge the exact function of ZNF441 has not yet been revealed, but the large family of transcriptional regulators of KRAB-containing zinc finger proteins are known to act as tumour suppressors [26]. In general, zinc finger proteins are a highly abundant group of proteins that varies in both structure and function [27]. They are involved in several cellular activities, including development, differentiation, and tumour suppression [28]. A zinc finger is a peptide domain whose secondary structure is stabilized by a bound zinc ion and a zinc finger protein can contain between 1 and 40 such domains [27]. The C2H2-domain is considered the “classical” zinc finger and is among the most abundent ones of the zink finger domains [28]. Zinc fingers were originally considered only as DNA-binding domains, but their role in protein-protein interactions has eventually been recognized [29]. Proteins with multiple zinc fingers can have two to three different types of binding activity through different fingers [28].

The Krueppel-associated box (KRAB-domain) is located near the N-terminal end of the protein, spans across 50–75 amino acids, and is divided into two boxes (A and B). KRAB-containing proteins are transcriptional repressors and use the zinc fingers to bind DNA [29]. KRAB-containing proteins are critical to cell differentiation, proliferation, apoptosis, and neoplastic transformation. Increased expression of the ZNF23 has been found to induce apoptosis in ovarian cancer cell lines [30]. ZNF431 functions as a transcriptional repressor for Patched1 (PTCH1) through binding to the target promoter sequence [31]. PTCH1 is a member of the Hedgehog (HH) family and acts as a negative regulator of the HH pathway. This pathway is important during embryonic development but has also been shown to be active during cancer development in adults. [32]. Repression of PTCH1 in a gastric cell line [33] was shown to correlate with high level of methylation of CpG islands at regulatory sequences and this could be associated with the development of gastric cancer. Another zinc finger protein, ZNF411, was found to suppress the MAP kinase signalling pathway [34], which is important for cell cycle checkpoints [35]. Overexpression of this pathway has been reported in different squamous cell carcinomas [36, 37]. The relationship between CIN grade and the MAP kinase pathway has also been investigated and was found to be an early marker for cervical carcinogenesis but not related to virus clearance [38]. Furthermore, the oncogenic E6 and E7, expressed in high risk HPV and known to play an important role in CIN tumour progression, also contain zinc finger domains, as recently reviewed by Ruttkay-Nedecky et al. [39]. In fact, new cell-permeable artificial zinc finger proteins (AZPs) have been launched as potential antiviral drug candidates that are able to reduce HPV replication [40, 41].

Phospholipase D6 (PLD6) was only reported by the CART analysis as contributing to the discrimination. In general, phospholipase D (PLD) proteins have been implicated in membrane trafficking [42, 43], cytoskeletal reorganization [44], endocytosis, exocytosis, cell migration, and cell proliferation [45]. The mouse homologue Zucchini (*mZuc*), also known as PLD6, has been shown to possess single strand-specific nuclease activity. This endoribonuclease has been shown to be essential for primary piRNA biogenesis [46, 47]. piRNAs are a distinct class of small RNAs, called Piwi-interacting RNAs, and have been discovered in both mammalian [48] and Drosophila germline [49]. They cluster at transposon loci in male germline stem cells and it has been suggested that piRNAs and their associated Piwi proteins are involved in epigenetic mechanisms like methylation and chromatin modifications [49]. A piRNA population has also been identified in the He-La cervical cancer cell line [50]. In germline stem cells these components are critical for silencing mobile genetic elements via DNA methylation. [51]. Furthermore, piRNAs have been detected in human cancer and somatic cells, and epigenetic disruption of the PIWI/piRNA pathway is indeed a hallmark for cancer development in testis [52]. Diminished piRNA expression has been found in testicular tumours as compared to normal testis.

In the current study, PLD6 was found to be expressed in most regression cases (12/20) but not in the persistent cases. The exact mechanisms for the epigenetic silencing exerted by the piRNA-PIWI pathway components remain unsolved and identification of additional protein components is crucial for a better understanding of the role of piRNAs in cancer [53].

This study and a previous study [17] show that CIN biopsies shed a complex mixture of proteins into a cell culture medium when placed at 4°C for 24 hours. For the LC-MS/MS study, supernatants from two series of 20 patient samples each (10 CIN2-3 with regression and 10 with persistence in each series) were analysed using a bottom-up shotgun proteomics approach [54] in which the proteins were digested into smaller peptides using trypsin. The peptide mixture was then analysed using unidimensional LC-MS/MS. Samples were pretreated by an immunoaffinity adsorbent which was previously validated by SDS-PAGE and LC-MS/MS (Supplementary Figure 1). Despite the depletion of seven high abundance proteins including immunoglobulins and albumin, peptides from these proteins were detected, while transferrin was not found at all after depletion. In addition, not unexpectedly, hemoglobins constitute a relatively large part of the identified proteins (cervical tissues with CIN2-3 are usually richly vascularised) and should be included in future depletion work. The complexity of the depleted fraction is still a challenge. Further fractionation of the depleted samples prior to the LC-MS/MS analysis would be an advantage to increase the supernatant proteome coverage and also possibly the sequence coverage of the identified proteins. This fractionation could be obtained using 2D-gel separation of the protein mixture or, for example, a cation exchange fractionation of the peptides after digestion. Another option is enrichment of subproteomes like phosphorylated proteins or glycosylated proteins. The results in this study should of course be validated by analysing a larger number of samples and also by analyses using other methodology like immunohistochemistry.

## 5. Conclusions

Using three different statistical methods to analyse normalized spectral count data, this study has identified zinc finger protein 441 as a highly discriminating factor between CIN2-3 regressive and persistent lesions. Phospholipase D6 contributes to the discrimination.

Interestingly the two proposed proteins are important factors for repression of tumour growth. Zinc finger proteins constitute the largest family of transcriptional regulators in mammals with important DNA binding domains and are also involved in protein-protein interactions. Their ability to induce apoptosis has been shown, as well as their function as nuclear transcriptional repressors of genes involved in signal transduction important for development of carcinogenesis. PLD6 is involved in biogenesis of piRNAs, small noncoding RNAs involved in hypermethylation events and important for transcriptional, epigenetic, and signalling pathways alterations. In line with these findings, both PLD6 and ZNF441 were almost absent in the persistent CIN2-3 cases contrary to the regression cases (Figure 4).

The depletion of seven high abundance proteins followed by a unidimensional separation of tryptic digests of nondepleted protein mixtures shows the potential of the described method for collection of proteins from CIN biopsies. From a prognostic aspect, the findings are promising tools for further investigation and understanding of the biology behind regression of precancerous cervical lesions.

## Supplementary Material

Excel spreadsheets containing peptide identification details for all proteins from the complete dataset with regression, table 1, and without regression, table 2

## Figures and Tables

**Table 1:** List of 165 proteins identified with high significance filter in Proteome Discoverer.

Accession number	Number of AAs	MW [Da]	Description	ΣCoverage
25777600	953	105769	26S proteasome non-ATPase regulatory subunit 1	1,1
92090990	757	86306	Activating signal cointegrator 1 complex subunit 2	1,7
119631843	438	48626	Activin A receptor, type I	7,3
90819233	1651	187556	Afadin isoform 2	1,5
112877	201	23497	Alpha-1-acid glycoprotein 1	15,9
119598593	366	39172	Alpha-2-HS-glycoprotein	5,7
224809474	951	106839	Ankycorbin isoform B	3,5
119573007	92	10573	Apolipoprotein A-II	52,2
119621207	3000	338317	Apolipoprotein B (including Ag(x) antigen)	0,8
73622085	665	72088	ATPase WRNIP1; AltName: Werner helicase-interacting protein 1	2,4
85700402	1704	191239	ATP-binding cassette subfamily A member 3	2,1
14916956	633	69181	ATP-dependent Clp protease ATP-binding subunit clpX-like, mitochondrial	3,2
260763963	652	73680	B-cell scaffold protein with ankyrin repeats isoform 3	2,8
410564	27	2891	Beta-trace {N-terminal}	74,1
119630319	616	69142	BTB and CNC homology 1, basic leucine zipper transcription factor 1	3,4
110618250	651	69567	Cadherin-related family member 5 isoform 3	2
93204551	754	85485	Calpain-7-like protein	2,3
119621019	2225	242829	Carbamoyl phosphate synthetase 2, aspartate transcarbamylase, and dihydroorotase	0,6
215274265	626	70812	Carnitine O-acetyltransferase	2,1
119588550	361	39391	CD44 antigen (Indian blood group)	3,3
151101301	550	60746	Centrosomal protein POC5 isoform 2	6,7
171184451	3117	350716	Centrosome-associated protein 350	0,9
283135365	21	2382	Chain A, human insulin	100
289526593	120	13375	Chain B, crystal structure of monomeric human cystatin C	9,2
296278495	120	13562	Chain B, crystal structure of the second bromodomain of human polybromo (protein polybromo-1)	14,2
295789308	153	15779	Chain B, human sod1 D124v variant	64,7
293651901	243	28061	Chain B, solution structure of double super helix model (apolipoprotein A-I)	33,3
289526844	220	24615	Chain B, structural basis of membrane-targeting by dock180 (dedicator of cytokinesis protein 1)	8,6
291463533	127	13753	Chain B, wild type human transthyretin (Ttr)	38,6
289526762	30	3428	Chain D, enhancing the therapeutic properties of a protein by a desig binding site	100
294979722	141	0	Chain E, deoxy human normal adult hemoglobin	56,7
294979723	146	0	Chain G, deoxy human normal adult hemoglobin	89
295321918	374	41579	Chain J, model of alpha-actinin Ch1 bound to F-actin	9,1
119630802	740	78794	Chromosome 20 open reading frame 75	2,7
119568512	153	17486	Chromosome 6 open reading frame 75	10,5
10518503	444	49288	Coagulation factor VII isoform B precursor	3,4
119581366	1261	135504	Cordon-bleu homolog (mouse)	2,3
83582815	89	9881	Cornifin-B	19,1
119602496	311	35475	Cysteine/histidine-rich 1	4,2
10719963	520	59956	Cytochrome P450 4F8	3,1
119581796	712	78265	Cytoplasmic polyadenylation element binding protein 4	2,4
119610301	2073	237519	Dedicator of cytokinesis 11	0,9
20141302	110	11277	Dermcidin	25,5
55749932	470	53503	Desmin	10,9
63054852	508	58371	DNA nucleotidylexotransferase isoform 2	5,3
119614620	977	109666	Endoplasmic reticulum to nucleus signalling 1	1,6
24418674	974	110429	Exocyst complex component 4	1,5
17369686	1087	123828	Exportin-7	1,5
22749363	434	49418	F-box only protein 15 isoform 1	3,5
119570461	1769	200655	Fer-1-like 3, myoferlin (*C. elegans*)	0,9
119568019	1322	142839	Fibronectin type III domain containing 1	1,5
13129018	188	20994	Gamma-glutamylcyclotransferase	10,1
119596338	147	16448	Ganglioside-induced differentiation-associated protein 1-like 1	10,2
46409304	443	48955	Glutamate-rich protein 1	3,8
119625129	215	24427	Glycoprotein M6A	7,4
119621332	947	107158	GREB1 protein	1,5
152031617	669	72408	GTP-binding protein 1	2,1
119625298	455	51153	Guanylate cyclase 1, soluble, alpha 3	2,9
119583777	947	106793	hCG1641824	2
119584015	69	7604	hCG1813122	17,4
119597394	4919	535890	hCG19253	0,4
119582778	87	10173	hCG1981126	12,6
119610862	1925	215078	hCG1986053	1,6
119568453	491	54322	hCG2030297	5,1
119572490	576	62385	hCG2040584	4,3
119598528	57	6808	hCG2045397	29,8
4504517	205	22768	Heat shock protein beta-1	21
119589211	147	16045	Hemoglobin, delta	62,6
119589125	462	51643	Hemopexin	7,6
121925	221	22336	Histone H1.3	5,9
5901922	378	44440	Hsp90 cochaperone Cdc37	3,7
164684901	94	11002	Immunoglobulin heavy chain variable region	18,1
17366467	2758	313745	Inositol 1,4,5-trisphosphate receptor type 1	0,6
119623087	1257	133685	Insulin receptor substrate 4	1
226694184	1179	130077	Integrin alpha-E	1,7
261878618	623	69452	Inter-alpha-trypsin inhibitor heavy chain H1 isoform C	2,3
27477074	502	55848	Interleukin-17 receptor B precursor	2,6
46397807	449	49715	Keratin, type I cuticular Ha7	3,3
195972866	584	58766	Keratin, type I cytoskeletal 10	22,6
239938886	623	62027	Keratin, type I cytoskeletal 9	28,3
238054406	644	65999	Keratin, type II cytoskeletal 1	30,1
239938650	639	65393	Keratin, type II cytoskeletal 2 epidermal	7,5
143811411	590	62340	Keratin, type II cytoskeletal 5	11,9
5031839	564	60008	Keratin, type II cytoskeletal 6A	14,2
238054404	564	60030	Keratin, type II cytoskeletal 6B	9
59803089	564	59988	Keratin, type II cytoskeletal 6C	16,3
90110027	483	53671	Keratin, type II cytoskeletal 8	7,9
119619543	916	102928	KIAA1024 protein	2,1
119600758	1047	116840	KIAA1128	4,3
119583893	1420	158295	Kinesin family member 13B	2,9
231569458	629	70939	Lactoperoxidase isoform 3 preproprotein	2,5
27436948	634	70618	Lamin-A/C isoform 3	8,2
119599090	222	25734	Latexin	5,4
119628276	394	43749	Mannosidase, alpha, class 1C, member 1	6,4
290457624	397	41734	Mesoderm posterior protein 2	4,8
115502451	496	53464	Mothers against decapentaplegic homolog 6	4
119573924	271	28644	Myeloid cell leukemia sequence 1 (BCL2-related)	7,4
119610415	1737	199228	Myosin, heavy polypeptide 8, skeletal muscle, perinatal	1,2
33667040	1048	119352	NACHT, LRR, and PYD domains-containing protein 8	2,7
33624861	429	48129	Nesprin-2 isoform 2	4
93204871	628	70024	Netrin-4 precursor	2,4
160332335	5890	628699	Neuroblast differentiation-associated protein AHNAK	4,5
119625194	527	60213	NIMA- (never in mitosis gene A-) related kinase 1	2,9
156632525	714	80686	Nuclear protein MDM1	2,4
119570426	800	92490	Nucleolar complex associated 3 homolog (*S. cerevisiae*)	1,6
74749412	510	57244	Olfactomedin-4	2,8
24430183	638	73293	Outer dense fiber protein 2 isoform 2	2,7
21735584	820	93729	Oxysterol-binding protein-related protein 3 isoform D	1,7
296439282	595	68541	P2X purinoceptor 7	2,2
237757297	643	70596	Pannexin-2 isoform 2	2
119626787	301	34291	Phosphatidylinositol glycan, class K	6,3
74730959	315	35934	PIH1 domain-containing protein 2	4,1
74730663	189	20681	Plasma cell-induced resident endoplasmic reticulum protein	18,5
296439496	1271	139580	Pleckstrin homology domain-containing family G member 4B	1,1
119602579	2105	233975	Plectin 1, intermediate filament binding protein 500 kDa	1
150421625	764	83232	Polymeric immunoglobulin receptor	2,8
123402	479	51177	POU domain, class 2, transcription factor 2	2,9
22261792	1499	167582	Probable phospholipid-transporting ATPase VA	3,7
32171249	190	21015	Prostaglandin-H2 D-isomerase	17,4
119578886	247	26680	Protease, serine, 3 (mesotrypsin)	5,3
122801	352	38974	Protein AMBP; AltName: Full = Alpha-1 microglycoprotein	6,5
162416266	758	87316	Protein dpy-19 homolog 2	1,7
257743264	606	69420	Protein THEMIS isoform 3	3,1
296452931	1208	138572	Protein timeless homolog	1,3
74733527	701	80649	Pseudouridylate synthase 7 homolog-like protein	2
167016536	107	12037	Putative nucleosome assembly protein 1-like 6	10,3
74760358	247	26522	Putative trypsin-6	12,2
74712786	109	11855	Putative uncharacterized protein FP588	18,4
119631914	1896	211398	RAP1 interacting factor homolog (yeast)	0,8
125987856	611	69368	Rho-related BTB domain-containing protein 3	2,5
119620511	39	4319	Ribosomal protein S27a	41
126215690	3280	373742	RING finger protein 213	0,7
296452978	1393	152659	RNA polymerase II-associated protein 1	0,9
119573716	93	10828	S100 calcium binding protein A8 (calgranulin A)	11,8
119573719	114	13234	S100 calcium binding protein A9 (calgranulin B)	28,1
4506027	307	35057	Serine/threonine-protein phosphatase 4 catalytic subunit	4,9
113576	609	69321	Serum albumin precursor	34,3
119569088	107	12318	SH3 domain binding glutamic acid-rich protein like 2	14
119576122	262	29185	Similar to CG12314 gene product	7,3
119620924	753	82996	Similar to RIKEN cDNA 4632412N22 gene	2,5
296452999	2785	318182	Small subunit processome component 20 homolog	0,9
119589494	452	50195	Solute carrier family 25 (mitochondrial carrier; phosphate carrier), member 23	4,4
119596056	314	33833	Solute carrier family 9 (sodium/hydrogen exchanger), member 8	4,1
119599799	450	51876	Sorting nexin 4	4,2
119628509	259	29439	Steroid-5-alpha-reductase, alpha polypeptide 1 (3-oxo-5 alpha-steroid delta 4-dehydrogenase alpha 1)	9,7
119599133	506	58721	TCDD-inducible poly(ADP-ribose) polymerase	2,2
109895218	526	57476	Thymocyte selection-associated high mobility group box protein TOX	6,1
291045225	33423	3711285	Titin isoform N2-A	0,3
119605952	641	71538	TNF receptor-associated factor 7	2,7
585328	80	8635	Trefoil factor 3	22,5
119573617	242	28050	Tropomyosin 3	14,5
14389309	449	49863	Tubulin alpha-1C chain	7,4
47157315	1124	125019	Tyrosine-protein kinase JAK3	1,3
55977767	466	53619	Vimentin	19,7
32483410	474	52883	Vitamin D-binding protein precursor	17,5
119568661	331	36884	WNT1 inducible signaling pathway protein 3	4,5
20532312	653	74145	Zinc finger protein 274	11,6
149588643	679	77891	Zinc finger protein 283	1,9
140560957	590	68217	Zinc finger protein 285A	2,4
30580627	626	72145	Zinc finger protein 441	2,2
74759403	364	41163	Zinc finger protein 589	4,7
119603081	647	74123	Zinc finger protein 595	2,5
119598822	485	56018	Zinc finger protein 639	2,5
187671927	394	46069	Zinc finger protein 763	3,8
74758703	808	93088	Zinc finger protein 841	2
